# International dissemination of evidence-based practice, open access and the IACAPAP textbook of child and adolescent mental health

**DOI:** 10.1186/s13034-015-0084-1

**Published:** 2015-11-10

**Authors:** Joseph M. Rey, Olayinka Olusola Omigbodun

**Affiliations:** Notre Dame School of Medicine Sydney, Sydney, Australia; Discipline of Psychiatry, Sydney Medical School, University of Sydney Medical School, Sydney, Australia; College of Medicine, University of Ibadan and Consultant in Child and Adolescent Psychiatry, University College Hospital, Ibadan, Nigeria

**Keywords:** Child Mental Health Education, Dissemination of evidence-based practice, e-books, Open Access Publishing

## Abstract

Dramatic changes have occurred in both publishing and teaching in the last 20 years stemming from the digital and Internet revolutions. Such changes are likely to grow exponentially in the near future aided by the trend to open access publishing. This revolution has challenged traditional publishing and teaching methods that—largely but not exclusively due to cost—are particularly relevant to professionals in low and middle income countries. The digital medium and the Internet offer boundless opportunities for teaching and training to people in disadvantaged regions. This article describes the development of the IACAPAP eTextbook of child and adolescent mental health, its use, accessibility, and potential impact on the international dissemination of evidence-based practice.

## Background

Amazon announced in May 2011 that its eBook sales in the US had exceeded its printed book sales. Changes in both publishing and teaching in the last 20 years stemming from the digital and Internet revolutions are turning on its head traditional publishing and teaching approaches. Conventional methods discriminated against professionals in low and middle income countries by making access to scientific information difficult due to the high cost of textbooks and journals and the lack of trained professionals to do the teaching and training—although important inroads are being made to overcome this, e.g., BioMed Central. Now, the digital medium and the Internet offer boundless opportunities for teaching and training people in disadvantaged economies. An electronic book (eBook, digital book) is a book-length publication in digital form, consisting of text, images, or both, that can be read on computers, tablets, smart phones or other electronic devices [1].

Among other objectives, the International Association for Child and Adolescent Psychiatry and Allied Professions (IACAPAP) [2, 3] aims to promote the study, treatment, care and prevention of mental and emotional disorders and disabilities of children, adolescents and their families. Few would question the urgent need for dissemination of optimal clinical practice and training in child and adolescent mental health but it is necessary to highlight the extent of this need and place IACAPAP’s efforts and specifically the IACAPAP eBook [4] in perspective. As Pang and her colleagues [5] wrote, not specifically referring to underprivileged populations, “Knowledge is the enemy of disease… Applying what we know already will have a bigger impact on health and disease than any drug or technology likely to be introduced in the next decade.” This is easier said than done, particularly in middle and low income countries, although health information is an essential component of many identified and established human rights [6]. An assessment of the progress made about the proposed goal of “universal access to essential health-care information by 2015” would be timely.

Important considerations are the quality of the information received and learned and how this information can be accessed. For example, in Pakistan, a cross-sectional survey of 1000 randomly selected general practitioners from urban areas found that almost half (40 %) used sedatives as their first line of treatment for hypertension and that the majority (63 %) relied on representatives from pharmaceutical companies for updates [7].

Another key factor is the availability of appropriately trained professionals. A study at the Kenyatta National Hospital (Kenya) found that more than half (56 %) of the 130 physicians working at that hospital expressed a need for further training to deal with psychiatric conditions in their patients [8]. Low and middle income countries have a severe shortage of health professionals—more so in psychiatry and particularly in child and adolescent mental health (CAMH). The World Health Organisation’s (WHO) *Child and Adolescent Mental Health Atlas* [9] reported that nowhere in the globe was the need for CAMH services fully met. Notably, countries with the highest proportion of children were the ones that lacked services the most. With few exceptions, African, Eastern Mediterranean, Southeast Asian, and Western Pacific countries had 1–4 child and adolescent psychiatrists per million population. In 2005, in the entire African continent outside of South Africa, fewer than 10 psychiatrists could be identified who were trained to work with children. Further, in these countries, social workers, psychologists, nurses and other professionals were not utilized for the mental health care of children and adolescents because of lack of training.

This article describes the development of the IACAPAP eBook of Child and Adolescent Mental Health and its potential impact on training and on the international dissemination of evidence-based practice. This information may be valuable for individuals and organisations interested in clinicians’ education.

## A child and adolescent mental health guide in every palm

In the 1970s, one of the authors (JMR)—then a recent medical graduate—owned a section of the *Encyclopédie Médico*-*Chirurgicale (EMC)* [10]. What made this reference book different is that a few chapters were updated every year for subscribers—although expensive, having the *EMC* is still a “must” for French medical practitioners (Jean-Philippe Reynaud MD, personal communication, November 13, 2013). The *EMC* is a good model to follow but, surprisingly, did not catch on in the English-speaking publishing world where the traditional textbook has remained the king, in spite of textbooks often becoming out of date, at least in part, by the time they are published given publication long lead time [3]. The advent of the Internet is gradually undermining the usefulness of reference textbooks because most clinicians, particularly younger ones, prefer consulting articles on line, the full text of a growing proportion of them being freely available.

“In 1998 I worked with paediatricians in Bucharest, Romania and then again in 2010 I did a rotation in Gaborone, Botswana. Both times, I left all my medical books behind, as they were a coveted and rare resource” (Julie Chilton MD, personal communication, December 13, 2013). In low and middle income countries, most students and professionals cannot afford to buy professional books and, at best, rely on using them in their institution’s library. Purchasing second hand, out-of-date editions or making photocopies are often the only alternative. Traditional textbooks also have the limitation that many topics do not need frequent updating—for example, those on historical and ethical aspects, assessment, and mental state examination—while others would benefit from incorporating new knowledge often, but the whole volume needs to be printed. Moreover, e-learning is increasingly seen as an important feature in training health professionals, especially in low and middle income countries [11]. If an eBook were available free and accessible online, it could be very useful.

## Would professionals in developing countries actually like using eBooks and have the means to access them?

Evidence suggests that to be the case. For example, a survey of 37 East African surgeons found that they preferred electronic journals to printed textbooks [12]. Another study found that smartphones were effectively utilized by resident physicians in resource-limited settings, both for accessing point-of-care medical information and for self-directed learning [13]. In 2000, people in developing countries owned one-fourth of the world’s 700 million mobile devices. By the beginning of 2009 this had grown to 3 billion [13]. According to Strategy Analytics, global smartphone users are expected to reach 2.5 billion by the end of 2015. Asia Pacific countries are estimated to account for the lion’s share of this, particularly due to growth in China, India, Indonesia, Philippines and Vietnam [14]. This suggests that digital delivery of medical information is not only welcomed by users but that the number of professionals accessing it is growing rapidly in low and middle income countries and that most clinicians already have a smartphone in their pocket.

## The eBook

Early in 2011, IACAPAP was approached with the idea of publishing an e-textbook that would be available free of charge under the terms of the Creative Commons Attribution Non-commercial License [15], which permits use, distribution, and reproduction in any medium, provided the original work is properly cited and use is non-commercial (the so called “Gold Open Access”). The rationale for providing open terms of both access and use is that free access offers the literature to students, clinicians, researchers, patients and their families whether they can afford to pay or not. Additionally, “granting readers full reuse rights unleashes the full range of human creativity for translating, combining, analysing, adapting, and preserving the scientific record” [16], thus multiplying the book’s impact. The book was also expected to involve contributors from low income countries to ensure that issues specific to their circumstances were taken into account and addressed. It was also planned that eBook chapters would be updated regularly and new chapters added to make it increasingly comprehensive.

The eBook would not have been possible without the generous contribution of a small army of experts (see list on the website) [4]. Since everyone involved contributed their work for free, the monetary cost of this project has been nil so far. The initial version of the textbook was made available in PDF format at IACAPAP’s website at the end of June 2012.

**Fig. 1:**
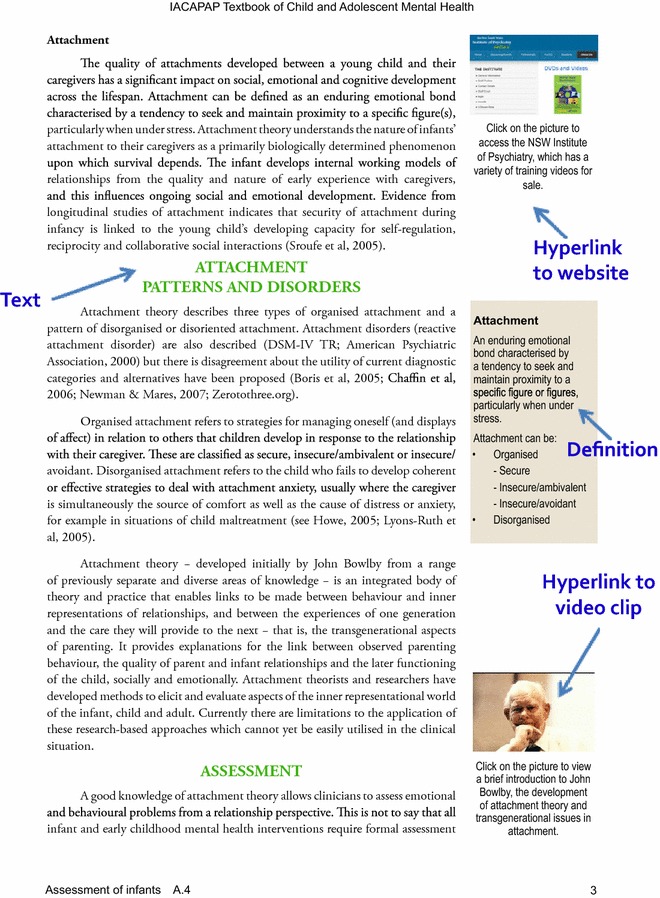
Example of a page layout

The eBook consisted initially of 42 chapters (more have been added since then) as well as an introductory section. Overall, the book had 940 pages initially. Contributors included 102 experts from 24 countries (USA: 15; Germany: 12, Brazil: 10; Canada: 10; UK: 9; Australia: 8; Malaysia: 6; Spain: 4; France: 3; Hong Kong: 3; Turkey: 3; China, Japan, the Netherlands, Nigeria, and Taiwan: 2; India, Kenya, Mexico, Namibia, Singapore, South Africa, Switzerland, and Tunisia: 1). There are dozens of video-clip links and hundreds of hyperlinks to original, freely available measuring instruments, websites and publications. Each chapter was organised as a separate entity so that it can be accessed and downloaded easily; this also allows replacement with a revised chapter without much disruption. However, there is no specific search facility; readers need to use the generic “find” tool available in Acrobat Reader. Figure 1 shows the layout of one of the pages.

Independent reviews have been largely positive: “…the book is a major contribution to international efforts to increase understanding regarding the diagnosis, treatment, and prevention of mental health problems in children and adolescents and a possible harbinger of future directions for more traditional textbooks” [17]. “Some striking features set this work apart from traditional textbooks. Most chapters contain embedded links to online resources such as academic papers, practice guidelines, government publications, public domain instruments, YouTube videos and other websites. For instance, in the chapter on tic disorders, a touch on the screen or a mouse-click takes the user directly to a video of children describing and exhibiting their tics—a resource that is not possible in the printed medium. The depression chapter has a hyperlink to the full 68-page *NICE Clinical Guideline on Depression in Children and Young People*. The autism chapter does not merely describe the theory of mind—it explains the concept by a video demonstration of an autistic child performing the ‘Sally-Anne test’. In the ADHD chapter, the user can instantly access online copies of symptom rating scales” [18].

## Have the objectives of the eBook been achieved?

Informal comments suggest the eBook is being used widely from Vilnius University to Yale University. “The textbook is attractively designed and easy to read. Because of the active sidebars, videos and links, studying is more fun and dynamic and readers are allowed to further expand and deepen their knowledge,” wrote two medical residents graduating in the child and adolescent psychiatry program at Vilnius University, Lithuania [19].

An important barrier to access, possibly underestimated in English-speaking countries, is lack of knowledge of English (e.g., in China, North African, Eastern European and South American countries). Individuals and organisations from several countries have expressed interest in translating the eBook—several chapters in French and Portuguese are already available, and work is being done in the translation into Russian, Spanish and Japanese.

## Outcomes

Measuring the usefulness of a book, electronic or otherwise, is a difficult task. How many copies are sold or how many people view a website are often used as outcome measures. Given the open nature of the eBook and its free access, monitoring outcomes is more challenging, among other reasons because it is not known how many people actually use it—not only by viewing the eBook on line but also by distributing it electronically or printing it—and for what purposes. It is of note that not using the eBook online deprives readers of many of its features such as immediate access to video clips.

In March 2015 a preliminary version of the international child mental health (iCAMH) curriculum was taught to 12 final year pediatric residents at Addis Ababa University by a local consultant child psychiatrist and a visiting child psychiatrist. The eBook was the main resource. As internet connectivity is sporadic and expensive in Ethiopia the relevant chapters were installed in a communal computer where participants could copy them for personal use. Student feedback on the material (from those who managed to read it) was very good. Chapters were seen as interesting and easy to understand. Especially in combination with face-to-face teaching, the material was found highly relevant clinically. Perhaps the greatest value of the IACAPAP textbook was that it served as excellent material from which the complete curriculum could be developed by the teachers (personal communication, Henrikje Klasen, 24 April 2015). That is, one of the main barriers for the eBook’s usefulness in low income countries is Internet access, consistent with the view that “If the goals of the draft declaration and action plan of the African Higher Education Summit [Dakar, 10–12 March 2015] are to be achieved, there should be less focus on building traditional universities and more on expanding high-speed broadband internet that will enable global cutting-edge knowledge to be delivered to students cost-effectively” [20].

Thus, the data available are anecdotal, relying on individuals who volunteer information and therefore likely to be positively biased. This said, we are keen on learning more about the book’s usefulness and discussions are taking place about finding ways to do that within the constraints in which this work is being done. An independent evaluation of the eBook using the OPAL framework (open educational practice maturity matrix) has been published by Coughlan and Perryman [21] of The Open University (UK).

## Readership

**Fig. 2:**
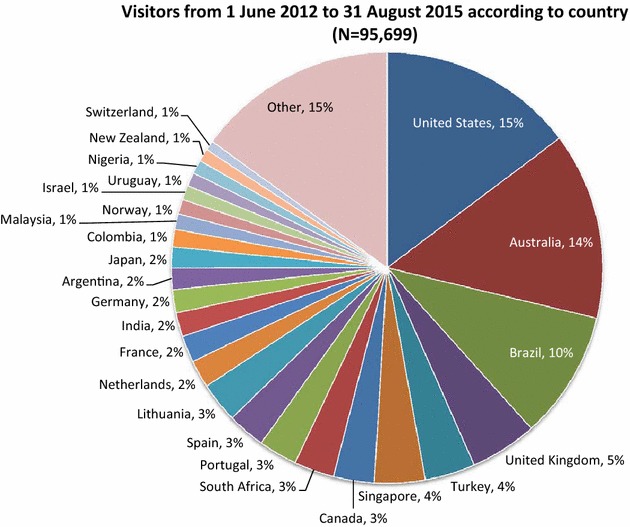
Proportion of pageviews (N = 95,699) to the eBook from July 1st, 2012 to August 31st, 2015 according to country of residence

The success of a book is largely reflected in the number of readers. From 1 June 2012 to 31 August 2015 there were 95,699 pageviews. The proportion of visitors to the textbook according to country is shown in Fig. 2. It is important to keep in mind when interpreting this figure that in many low income countries the number of child mental health professionals can be counted with the fingers of one hand.

## Updating

One of the challenges in this type of publication is to maintain the enthusiasm and actually deliver in the demanding areas of regularly updating the content and adding new content to fill gaps. In this line, three new chapters were added in 2013: on DSM-5 (written by a Brazilian/USA team), e-therapy (by a New Zealand team), and HIV/AIDS (by a US/South African group). In 2014 the following chapters were added: case formulation and integration of information in child and adolescent mental health (by a US team); diagnosis and treatment planning in child and adolescent mental health (by an Australian); acute and chronic reactions to trauma in children and adolescents (by a French/US team). The chapter on autism spectrum disorders was updated (by a Spanish/US group). Several more chapters are to be revised and a few new chapters added in 2015.

## Readers’ participation

Interactivity may be enhanced by allowing readers to ask questions, make comments and optimally by being able to share these with other readers resulting in a more dynamic, satisfying and powerful learning experience. To facilitate readers’ interaction and involvement, a Facebook page exclusively dedicated to the Textbook was set up in January 2014. It was hoped that such a facility would enable readers to interact with each other, with the editor and contributors, as well as making comments and suggestions and receive textbook-related news [22]. So far, the success of this has been limited.

## The future

Apart from updating the current chapters and expanding the content with more chapters, the goal is to increase the teaching potential and interactivity to assist people providing training on the ground [23]. For example, self-directed learning power can be expanded by including exercises, self-assessment activities such as multiple choice questions, PowerPoint slides, and other practical activities. Some of these features have been gradually introduced since 2014 and are to be developed further.

## Abbreviations

IACAPAP: International Association for Child and Adolescent Psychiatry and Allied Professions
CAMH: child and adolescent mental health
EMC: Encyclopédie Médico-Chirurgicale
WHO: World Health Organisation
